# Cancer Prevention Pathways in People Living with HIV: Assessment of Prevalence and Related Factors Among Individuals Attending HIV Division of Ferrara Hospital

**DOI:** 10.3390/jpm15090434

**Published:** 2025-09-09

**Authors:** Daniela Segala, Mario Stancanelli, Rosario Cultrera

**Affiliations:** 1 Department of Translational Medicine and for Romagna, Azienda Ospedaliero Universitaria di Ferrara, University of Ferrara, 44121 Ferrara, Italy; 2 University of Ferrara, 44121 Ferrara, Italy; mario.stancanelli@edu.unife.it; 3 Department of Translational Medicine and for Romagna, Azienda Unità Sanitaria Locale di Ferrara, University of Ferrara, 44121 Ferrara, Italy; rosario.cultrera@unife.it

**Keywords:** human immunodeficiency virus infection (HIV), people living with HIV (PLWH), HIV-associated malignancies, cancer risk in HIV, HIV-related cancer prevention

## Abstract

**Background.** Oncological diseases are among the leading causes of death in people living with HIV (PLWH). With the introduction of antiretroviral therapy and the consequent reduction in AIDS-defining cancers (ADC), there has been a growing incidence of non-AIDS-defining cancers (NADC). **Methods.** A retrospective observational study (cross sectional prevalence analysis) was conducted to investigate the prevalence and spectrum of oncological diseases in patients attending the HIV/AIDS Division at the Ferrara Hospital. The sample included 534 patients evaluated between January 2023 and November 2024 (534/682 met eligibility). Demographic, clinical, and serological data were extracted from medical records. The CDC’s 2014 definition has been adopted for the ADC/NADC classification. Statistical analysis was performed using SPSS version 29 and G*Power 3.1 software. **Results.** The data analysis revealed 62.8% NADC vs. 37.2% ADC (44 NADCs vs. 26 ADCs). Male individuals and those aged 50 and older were more represented. Patients with ADC more often fell into C2–C3 groups, indicative of severe immunodeficiency, while NADCs were more prevalent in clinical groups A1–B3. Statistical analysis showed that viral load was more frequently under 50 copies/mL in the NADC group, while it tended to be higher in the ADC group. **Conclusions.** These results align with current scientific evidence regarding the global prevalence of ADCs and NADCs. The findings highlight the need to implement targeted oncological screening strategies for HIV-positive patients to promote early diagnosis and improve prognosis.

## 1. Introduction

Human immunodeficiency virus (HIV) infection continues to be a major global public health issue. In the early stages of the epidemic, the occurrence of cancer in people living with HIV (PLWH) was frequent and led to the definition of a subgroup of neoplasms called AIDS-defining cancers (ADC), including Kaposi’s sarcoma, non-Hodgkin lymphoma, and cervical cancer. The introduction of highly active antiretroviral therapy (HAART) significantly changed the clinical course of HIV, drastically reducing the incidence of ADCs, increasing the life expectancy of HIV-positive patients, and leading to a parallel rise in non-AIDS-defining cancers (NADC) [1,2]. The shift in the types of oncological pathologies is a global trend and has also been observed in the province of Ferrara, as a direct consequence of the success of modern HIV treatment. Several studies suggest a potential direct oncogenic role of HIV through chronic systemic inflammation and immune activation [3,4]. Moreover, coinfection with other viruses (e.g., HBV, HCV, HPV, EBV) may contribute to cancer development in PLWH [5]. This led to a retrospective observational study of the cohort of PLWH attending the HIV/AIDS Division at the Ferrara Hospital, aiming to investigate the incidence and spectrum of oncological diseases, categorizing them as ADCs and NADCs, and to explore potential correlations with the duration and severity of HIV, viral suppression, antiretroviral therapy, and the presence of traditionally recognized risk factors, including tobacco use, alcohol consumption, and coinfections (hepatitis viruses, herpesviruses, human papillomavirus, Treponema pallidum, and Toxoplasma gondii). Ferrara Province has never published cancer-screening data in PLWH: this research would therefore constitute the first publication of its kind for the area. Regional healthcare policies and public health initiatives designed to serve this population rely on fragmented information gathered from individual hospital-based registries. This reliance presents a major challenge because these internal hospital records may not provide a complete or accurate picture of screening practices across the entire PLWH community, hindering the ability to effectively monitor progress, identify specific needs, and implement targeted improvements in care. This underscores the critical importance of a personalized medicine approach, where screening and treatment strategies are tailored to the individual’s unique clinical and risk profile.

## 2. Materials and Methods

### 2.1. Study Design

**Figure A1:**
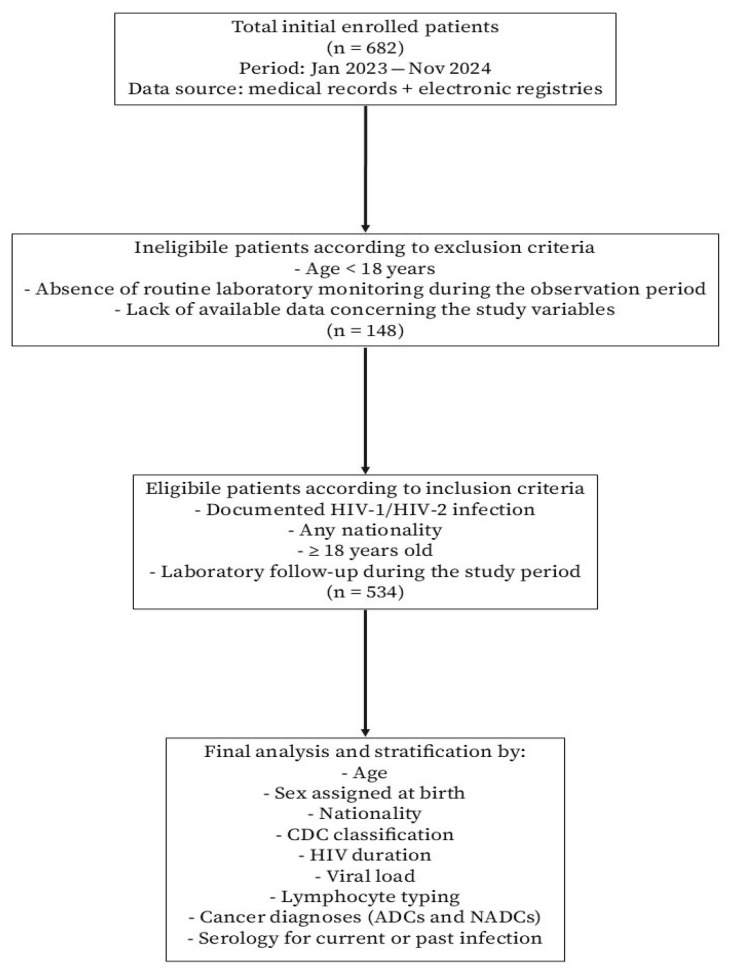
Study Design and Enrollment Flowchart.

A retrospective observational study (cross sectional prevalence analysis) was conducted on PLWH attending the HIV/AIDS Division at Ferrara Hospital, Italy, with an observation period between January 2023 and November 2024, covering a total duration of 23 months (Figure A1). The Center provides care for patients from the entire province of Ferrara, as well as for those from other cities and regions who choose to receive treatment here. The number of new patients annually has averaged around 50 in recent years, split between transfers from other centers and new diagnoses. The Center adheres to regional, national, and international guidelines (EACS, WHO) for the management of people living with HIV and the prevention of the infection. It also collaborates closely with the departments of oncology, hematology, surgery, diagnostic imaging, and laboratory services of the Ferrara Hospital for the diagnosis, treatment, and follow-up of PLWH who have oncological pathologies. This close collaboration is crucial for providing comprehensive, high-quality care. It ensures that the complex medical needs of people with HIV and cancer are met effectively. The goal of the study was to evaluate the prevalence of cancers, classified into ADCs and NADCs, and their association with demographic, clinical, virological, immunological, and behavioral variables.

### 2.2. Study Objectives:

• To assess the prevalence of oncological diseases among PLWH attending at the HIV/AIDS Division, in Ferrara.
• To evaluate the prevalence of ADCs and NADCs among patients with cancer diagnosis.
• To examine the correlation between the occurrence of ADCs and NADCs and variables such as age, sex assigned at birth, CDC clinical-prognostic classification, duration of HIV infection, antiretroviral therapy, HIV viral load, lymphocyte subset analysis, serological markers, and lifestyle-related risk factors (e.g., smoking habits, alcohol consumption, and drug use).

### 2.3. Inclusion Criteria:

• Age ≥ 18 years
• Documented diagnosis of HIV-1 or HIV-2
• Any nationality
• Laboratory follow-up during the study period

### 2.4. Exclusion Criteria:

Age < 18 years

Absence of routine laboratory monitoring during the observation period

Lack of available data concerning the study variables

### 2.5. Data Collection and Analysis

Data were extracted from clinical records and digitized into a database. Information included age, sex assigned at birth, nationality, CDC classification, HIV duration, antiretroviral therapy (drug classes), viral load, lymphocyte typing, cancer diagnoses (ADCs and NADCs throughout the patient’s entire period of affiliation with the Center), and serology for current or past infections by hepatitis viruses, herpesviruses, high-risk HPV, Toxoplasma gondii, and Treponema pallidum. Information on smoking habits, alcohol consumption, and drug use was self-reported by the patients. Plasma HIV RNA levels were quantified from venous blood samples using the Real-Time PCR assay routinely performed at the Provincial Central Laboratory of Ferrara Hospital (cut-off 20 copies/mL). Lymphocyte subset typing and serological testing were also conducted at the same laboratory, in accordance with its standard procedures. Instrumental diagnostics and imaging studies were performed at the Radiology department using ordinary methodologies. The continuous variable viral load was recoded as a dichotomous variable using a cut-off of 50 copies/mL (<50 copies/mL: virological suppression; ≥50 copies/mL: lack of suppression), whereas CD4+ T lymphocyte count was classified into three categories (>500 cells/μL: immunological stabilization post-ARV; <200 cells/μL: poor immunological control; intermediate values: partial response). Latency between HIV diagnosis and ADC onset was expressed as median (IQR) due to skewed data distribution, whereas latency for NADCs was expressed as mean (±SD) given the approximately normal and homogeneous distribution. Statistical analysis was performed using standard software (SPSS version 29 and G*Power 3.1), employing Fisher’s exact test and Chi-square test to compare groups.

## 3. Results

Of the 682 patients initially enrolled, the analysis was conducted on 534 individuals (78%), as 148 patients (22%) were deemed ineligible due to missing laboratory data, either because they were lost to follow-up or failed to attend scheduled follow-up visits. The analysis revealed a marked predominance of male individuals 68.5% (366/534), and those over the age of fifty 72.8% (389/534) with a mean age of 56 years and a median of 58 years. 58.9% (314/534) of patients had been living with HIV for less than 20 years, while the remaining 41.1% (220/534) had lived with it for 20 years or more. Virologically, 93.6% (500/534) of patients had a viral load below 50 copies/mL at their last follow-up, indicating good infection control; 77.5% (414/534) had a CD4+ lymphocyte count above 500/μL, further indicating immunological stabilization due to antiretroviral therapy. However, despite good viro-immunological control, 11.7% (63/534) of the sample had a cancer diagnosis (Table A1). Of these cancer diagnosis, 37.1% (26/70) had AIDS-defining cancers and 69.2% (44/70) had non-AIDS-defining cancers (Table A2). ADC cases included Kaposi’s sarcoma 27.1% (19/70), non-Hodgkin lymphoma 8.5% (6/70), and cervical cancer 1.4% (1/70). NADCs mainly consisted of skin neoplasms 20% (14/70), head and neck cancers 12.8% (9/70), thoracic tumors 2.8% (2/70), breast cancers 10% (7/70), urogenital tumors 8.5% (6/70), gastrointestinal cancers 5.7% (4/70), and hematolymphoid system neoplasms 2.8% (2/70) (Table A3). An overview of the cancers addressed in this study, classified according to ICD-10 codes, is provided in Table A4. Among cancer patients, there was again a strong male predominance 74.6% (47/63) and a majority aged over fifty 85.7% (54/63). However, among cancer cases occurring in individuals over fifty years of age (87.2%, 61/70), NADCs were more common (67.2%, 41/61); while ADCs (9/70) were proportionally more often diagnosed in younger patients (66%, 6/9). ADCs were associated with detectable viral load (>50 copies/mL) and lower CD4+ counts, reinforcing their link with advanced immunodeficiency. Conversely, NADC patients often had suppressed viral load (<50 copies/mL) and CD4+ counts >500/μL, suggesting these cancers may develop independently of severe immunosuppression. Among the 70 cancer cases analyzed, serological data were available for 55 patients: in these cases, seropositivity was indicative of either current or past infection. Serological profile analysis revealed significant coinfections: among NADC patients, seropositivity for HBV and HCV was observed in 75% (15/20) and 70.6% (12/17) of cases, respectively, higher than in the ADC group (25%, 5/20 for HBV; 29.4%, 5/17 for HCV). Regarding herpesvirus infections, HHV-1 and CMV were more prevalent in NADC patients (66.7%, 12/18 and 61.6%, 16/26, respectively) compared to those with ADCs (33.3%, 6/18 for HHV-1; and 38.4%, 10/26 for CMV). In contrast, EBV and Toxoplasma gondii showed similar prevalence across both groups. Exceptions to this pattern included HHV-2, HHV-3, and Treponema pallidum, which were more frequently detected in ADC patients: HHV-2 in 62.5% (5/8), HHV-3 in 71.4% (5/7), and Treponema pallidum in 64.7% (11/17). Subgroup analysis showed significant differences in latency between HIV and cancer diagnoses: ADCs tended to appear earlier (average latency of 8 years), while NADCs had an average latency of 17 years, suggesting a link with aging and chronic inflammation rather than active viremia. Regarding CDC classification, last revised in 2014, ADC patients were more often in classes C2 and C3 (advanced immunodeficiency), while NADCs were more frequent in categories A1–B3. This supports the idea that NADCs may develop independently of HIV progression stage. Statistical analysis (Table A5) confirmed a significant association between high viral load and ADCs (*p* < 0.05), while viral suppression was linked to NADC occurrence. However, in some cases, small sample sizes limited the applicability of the Chi-square test. Although combining categories was considered, it was not always methodologically justified. Despite this, the results clearly indicate two distinct clinical patterns in cancer patients: one strongly associated with immunosuppression (ADC) and another with non-immunological cumulative factors (NADC). Epidemiologically, among Italian men with ADCs, Kaposi’s sarcoma was most common (57.6%, 15/26), followed by non-Hodgkin lymphoma (15.5%, 4/26). Among all patients with ADCs, cervical cancer was detected in only one case, involving a woman of foreign origin (3.8%, 1/26). For NADCs, skin, head and neck, gastrointestinal, and urogenital cancers were predominant in men, whereas 7/44 cases (15.8%) of breast cancer were identified among women. Skin tumors were identified in patients with a history of oncological disease—either diagnosed prior to or following the diagnosis of an ADC or NADC: specifically, among these individuals, skin cancers were observed in 10.5% (4/38) of NADC patients and 3.8% (1/26) of ADC patients. In conclusion, these results confirm the effectiveness of antiretroviral therapy in reducing AIDS-defining cancers but also highlight the rising incidence of non-AIDS-defining cancers. This trend reflects the evolving clinical profile of an aging HIV-positive population and underscores the need for broader, inclusive, and specific oncological surveillance strategies for these patients.

## 4. Discussion

HIV infection represents one of the most significant global health challenges of recent decades, and if left untreated, it can progress to AIDS, an advanced clinical condition characterized by a critically low number of CD4+ T cells and the emergence of severe AIDS-related illnesses, including Kaposi’s sarcoma and certain forms of lymphoma. Our study provides compelling evidence for the urgent need to adopt a personalized medicine approach in the comprehensive care of PLWH. The traditional focus on AIDS-defining cancers is no longer sufficient. This evolving reality demands a holistic and tailored strategy that moves beyond generic screening guidelines. While standard protocols are a good starting point, they fail to account for the unique and multifaceted risk pro-files of individual PLWH. By leveraging key clinical data, including patient age, immune status, viral load, and the presence of coinfections like HBV and HCV, we can precisely identify individuals at elevated risk for specific malignancies. This granular understanding is crucial because it allows for the development of customized cancer prevention pathways that are far more effective than a one-size-fits-all approach. These personalized pathways would ensure that screening for specific conditions is prioritized for the patients who need it most. The introduction of the Highly Active Antiretroviral Therapy has radically transformed the clinical landscape, leading to a marked decline in the prevalence of AIDS-related diseases and a significant increase in life expectancy among HIV-positive individuals. However, this progress has contributed to a rise in the prevalence of malignancies, particularly non-AIDS-defining cancers, which now account for an increasing share of the cancer burden among PLWH. The most recent epidemiological data, including those collected in our study, confirm a well-established trend in the scientific literature: non-AIDS-defining cancers are now more frequent than AIDS-defining cancers, both in absolute and relative terms [6,7]. This phenomenon can be attributed to multiple factors: on one hand, improved survival due to HAART efficacy exposes patients to a lifespan long enough for malignancies with longer latency periods to develop; on the other, the prolonged state of chronic inflammation and immunosenescence, even in the presence of virological suppression, may play a key role in promoting malignant transformation [8]. In our sample, the combined prevalence of ADCs and NADCs was 11.7% (63/534), a figure in line with Western estimates but significantly higher compared to the HIV-negative population [9,10]. In relation to the international literature, our data are consistent with findings by Cattelan et al. (2023), who reported a 38.3% higher prevalence of NADCs compared to ADCs, and with observations by Hernández-Ramírez et al. (2017), according to whom PLWH have a 69% increased cancer risk compared to the general population [11,12]. The analysis of the sample’s demographic characteristics reveals additional noteworthy elements: patients with an oncological diagnosis were predominantly male and over 50 years of age. This reflects the aging process of PLWH in high-income settings, where access to care ensures good long-term quality of life. Aging is known to be one of the main risk factors for cancer, due to the accumulation of somatic mutations and a reduction in immune surveillance efficiency [13]. In our study, ADC cases were more common among individuals with detectable viremia and CD4+ counts below 200/μL, confirming the key role of immune compromise in the pathogenesis of these malignancies. The ADCs observed were mainly Kaposi’s sarcoma, non-Hodgkin lymphoma, and cervical cancer, in line with historical definitions and trends described by Moran et al. (2022) [14]. Furthermore, over half of ADC cases were diagnosed concurrently with HIV, suggesting that these cancers may be considered sentinel clinical manifestations of advanced infection. Conversely, NADCs tended to affect patients with suppressed viral load and CD4+ levels >500/μL. This suggests a pathogenesis independent of the severity of immunodeficiency, potentially mediated by alternative mechanisms such as chronic systemic inflammation, coexisting viral coinfections, and exposure to environmental or behavioral risk factors. In fact, our study found a higher prevalence of HBV and HCV seropositivity among patients with NADCs compared to those with ADCs, reinforcing the well-established link between these infections and hepatocellular carcinoma [15]. The distribution of NADC tumors in our sample showed a predominance of cutaneous, head and neck, breast, thoracic, gastrointestinal, and genitourinary cancers. The similarity between the oncological spectrum of HIV-positive and HIV-negative individuals underscores the need to fully integrate PLWH into cancer screening and prevention programs designed for the general population. Another relevant finding concerns the prevalence of skin cancers among patients with a prior cancer diagnosis: specifically, 10.5% of patients with NADCs and 3.8% with ADCs. Given the potential aggressiveness of such diseases in immunocompromised individuals, this finding suggests the usefulness of systematically introducing dermatological screening programs into HIV follow-up protocols to enable early identification of pre-neoplastic or neoplastic lesions with aggressive potential. The analysis of the relationship between gender and cancer incidence confirmed a male predominance, especially for NADCs. Although the distribution of cancer types differs between men and women (with lung and liver cancers more common in men, and breast cancers in women), the greater male cancer burden could reflect a combination of biological, behavioral, and healthcare access factors [16,17]. Another explanatory factor could be the more frequent late diagnosis of HIV in men, as highlighted by Arantes et al. (2023) [18]. The evaluation of the latency between HIV diagnosis and cancer onset showed a longer average time for NADCs compared to ADCs, further confirming the correlation between chronic infection and the development of non-AIDS-defining tumors. Also, the analysis of tumor-type distribution patterns reveals a substantial overlap with those observed in the HIV-negative population, suggesting that HIV infection does not act as a direct etiological factor in the development of non-AIDS-defining cancers, but rather functions as an accelerating or facilitating agent in oncogenic processes that are already present or latent, thereby contributing to the earlier onset of such malignancies in HIV-positive individuals, aligning with observations reported by Bray et al. (2024), regarding global cancer trends in 2022, and by Proulx et al. (2022), which outlines the molecular mechanisms underlying the acceleration development of NADCs [19,20]. According to the latter study, transferable HIV proteins, that can be delivered from infected cells to oncovirus-target cells through different mechanisms, including release (Env), diffusion (Tat), or exome/conduit-mediated assistance (Nef), could accelerate oncovirus-mediated tumorigenesis. Nevertheless, the mechanisms of cell-to-cell transfer of other HIV proteins, such as Rev and Vpr, remain still unclear. In addition, viral replication, coinfections, and the activity of HIV-1 proteins can profoundly alter immune and inflammatory responses within the tumor microenvironment, fostering survival signals and tumor growth. Finally, HIV-1 proteins can modulate gene expression, enhancing oncogenic factors and interfering with cellular signaling pathways involved in cell cycle regulation, proliferation, migration, inflammation and survival, thereby further promoting tumorigenesis. These findings, along with the higher prevalence of NADCs in individuals over 50, support the hypothesis that HIV is an indirect risk factor for cancer development, through mechanisms related to aging and the persistently inflammatory microenvironment. One final aspect deserves attention: the still unclear role of HAART in cancer pathogenesis [21]. Although there is no solid evidence supporting a direct oncogenic potential of these drugs, it cannot be ruled out that some compounds, in combination with individual and environmental factors, may contribute indirectly to carcinogenesis. Nevertheless, the overall benefit of HAART in terms of survival and prevention of ADCs remains indisputable. In conclusion, despite some limitations such as survivorship bias and missing data, the findings from our study reinforce the notion of a shifting cancer landscape among PLWH, where NADCs predominate in incidence and represent a new clinical priority. The 2024 update of the European AIDS Clinical Society (EACS) guidelines outlines targeted prevention protocols for the early diagnosis of anal, breast, cervical, colorectal, hepatocellular, prostate and lung cancers. However, several studies indicate that psychological and emotional factors related to the stigma associated with HIV infection, together with socio-economic barriers, may substantially limit the adherence of PLWH to the recommended screening programs [22,23]. Cancer prevention strategies in HIV-positive patients must necessarily adapt to this new reality, including targeted screening programs, greater attention to coinfections, and a multidisciplinary approach that integrates oncological management with immunovirological monitoring. Access to specialized services from diagnostic imaging and laboratory departments is essential. HIV-related cancers can sometimes present atypically and distinguishing them from other conditions requires specific expertise. This multi-departmental collaboration creates a unified, multidisciplinary team that can address both conditions at the same time, ensuring that patients undergo the most appropriate and advanced diagnostic tests to get an accurate and timely diagnosis. Given these premises, it is essential to promote service inclusivity, combat stigma, and improve health education to ensure equitable and timely access to diagnosis and treatment, thus guaranteeing better health outcomes and quality of life for this vulnerable population. Our findings support a proactive operational model, as outlined in our proposed cancer prevention pathways. A cornerstone of this approach is the seamless integration of oncological screening into routine HIV care, which is centrally managed by local HIV/AIDS Divisions. In this context, the term Cancer Prevention Pathways refers to the implementation of personalized, integrated and easily accessible clinical pathways for PLWH, designed according to individual clinical needs and exposure to established oncological risk factors, such as smoking, excessive alcohol consumption, diabetes and coinfections with oncoviruses. These pathways are managed directly by local HIV/AIDS Divisions in close collaboration with hospital specialty departments, with the aim of preventing cancer-related complications and improving overall clinical outcomes. A key feature of this model is that patients are not required to independently schedule specialist consultations. Instead of placing the burden on patients to navigate multiple hospital departments, this model ensures that the healthcare system takes the lead. HIV/AIDS Divisions proactively contact eligible individuals by phone to coordinate appointments dates with the relevant departments. This organizational approach not only facilitates greater adherence to existing cancer screening programs but also helps mitigate barriers related to the persistent stigma associated with HIV status. In fact, it can be a powerful deterrent, leading patients to avoid seeking care in new or un-familiar settings like oncology clinics. By keeping care within the established and trusted relationship with their HIV team, this model creates a safer and more familiar entry point for critical screenings. It also addresses socioeconomic barriers by reducing the logistical and financial burden on patients. This multidisciplinary collaboration, between immunovirologists, oncologists, dermatologists, and other specialists, is essential for ensuring that patients receive timely, coordinated, and comprehensive care. A critical next step for improving healthcare in Ferrara, and more broadly, throughout the region, would be the creation of a centralized regional or national registry of cancers occurrences in PLWH. The current reliance on fragmented, individual hospital-based records makes it nearly impossible to get a clear and complete picture of the health needs and screening practices of this community. A unified, consolidated dataset would be vital for public health officials. This data would allow them to pinpoint exactly where and how to implement specialized screening protocols specifically tailored to the PLWH population. With accurate information, healthcare providers could create and promote programs that encourage earlier and more consistent cancer screening among this group, ultimately leading to earlier diagnoses. Furthermore, a comprehensive registry would provide the necessary tools to effectively monitor these screening programs over time, ensuring that public health initiatives are meeting their goal of improving patient outcomes. A unified registry, with completely anonymous data analyzed in an aggregate manner, would overcome these limitations by providing a single, comprehensive data source. Ultimately, this shift toward precision prevention is about more than just early diagnosis; it is about fundamentally transforming the quality and longevity of life for PLWH. It is an actionable plan that builds upon the success of HAART by addressing the next frontier in HIV care. The proposed creation of a centralized, anonymous registry of cancers in PLWH would be a vital tool to make this personalized approach scalable and evidence-based, allowing public health officials to monitor outcomes and refine protocols over time. Our study serves as a critical step in this direction, providing the data necessary to move from a reactive treatment model to a proactive, patient-centered, and preventive one.

## 5. Conclusions

This study confirms the effectiveness of antiretroviral therapy in reducing AIDS-defining cancers, while highlighting the growing prevalence of non-AIDS-defining cancers in PLWH. These findings reflect the impact of longer survival, exposing patients to cancer risk factors common in the general population, exacerbated by immunosenescence and coinfections, which plausibly contribute. There is a pressing need to develop cancer screening strategies specifically for PLWH to promote early diagnosis and improve prognosis. However, the complexity of diagnostic and therapeutic pathways involving multiple specialists can be challenging for patients, particularly due to ongoing stigma associated with HIV. This research, as the first publication of its kind for the Ferrara area, would be a highly significant contribution to the field of public health, especially in the local context. Based on this work, the Center is currently investigating structured and facilitated screening pathways for major cancer conditions in people living with HIV who receive care at the facility, in collaboration with other hospital and community services. Creating accessible, integrated, and personalized screening pathways is a key strategy to overcome these barriers, accelerate early cancer detection, and significantly improve clinical outcomes for the HIV-positive population. Ultimately, a unified registry of oncological pathologies (with anonymous data) would move the healthcare system from a reactive, fragmented approach to a proactive, evidence-based strategy, leading to more effective early cancer detection and improved care for the entire PLWH community. In the context of personalized medicine, this study is a vital step forward. The findings underscore the need to move beyond a one-size-fits-all approach to healthcare. By creating structured and personalized cancer screening pathways, this research recognizes the unique biological vulnerabilities, such as immunosenescence and coinfections, and the psychosocial challenges, like stigma, faced by people living with HIV. This proactive, tailored strategy is crucial for optimizing early diagnosis and significantly improving both the quality of life and clinical outcomes for each individual patient.

## Data Availability

All authors have read and agreed to the submitted version of the manuscript. This work has not been presented previously at any meeting. The anonymized dataset used in this study is available upon reasonable request.

## Appendix A

**Table A1:** Descriptive analysis of the sample.

Criteria	No. Patients/Total (%)
Total Males Females	534 (100%) 366/534 (68.5%) 168/534 (31.5%)
Italians Males Females	443/534 (83%) 328/534 (61.4%) 115/534 (38.6%)
Foreign nationals Males Females	91/534 (17%) 38/534 (7.1%) 53/534 (9.9%)
Age < 50 years Age ≥ 50 years	146/534 (27.2%) 389/534 (72.8%)
Duration of HIV ≥ 20 years Duration of HIV infection < 20 years	220/534 (41.1%) 314/534 (58.9%)
CDC A1 CDC A2 CDC A3 CDC B1 CDC B2 CDC B3 CDC C1 CDC C2 CDC C3	39/534 (7.3%) 169/534 (31.6%) 33/534 (6.1%) 11/534 (2%) 81/534 (15.1%) 100/534 (18.7%) 1/534 (0.1%) 24/534 (4.4%) 76/534 (14.7%)
Viral load < 50 copies/mL Viral load ≥ 50 copies/ml	500/534 (93.6%) 34/534 (6.4%)
CD4 > 500/μL CD4 200–500/μL CD4 < 200/μl	414/534 (77.5%) 99/534 (18.5%) 21/534 (4%)
Serological history Available anamnestic data Unavailable anamnestic data	403/534 (75.4%) 131/534 (24.6%)
Ever-positive serology HBV HCV HHV-1 HHV-2 HHV-3 HHV-8 EBV CMV HPV-HR Toxoplasma gondii Treponema pallidum	122/403 (30.2%) 157/403 (38.9%) 105/403 (26%) 53/403 (13.1%) 38/403 (9.4%) 19/403 (4.7%) 141/403 (34.9%) 181/403 (44.9%) 11/403 (2.7%) 88/403 (21.8%) 107/403 (26.5%)

**Table A2:** Clinical characteristics of patients with AIDS-defining (ADC) and non-AIDS-defining (NADC) neoplasms.

Criteria	No. Patients/Total (%)
Total patients with cancer Total patients without cancer	63/534 (11.7%) 471/534 (88.3%)
**Criteria**	**No. Patients/Total**
Males Females	47/63 (74.6%) 16/63 (25.4%)
Italians Males Females	54/63 (85.7%) 43/63 (68.2%) 11/63 (17.4%)
Foreign nationals Males Females	9/63 (14.2%) 4/63 (6.3%) 5/63 (7.9%)
Age < 50 years Age ≥ 50 years	9/63 (14.3%) 54/63 (85.7%)
Duration of HIV infection ≥ 20 years Duration of HIV infection < 20 years	27/63 (42.9%) 36/63 (57.1%)
CDC A1 CDC A2 CDC A3 CDC B1 CDC B2 CDC B3 CDC C1 CDC C2 CDC C3	3/63 (4.7%) 7/63 (11.1%) 2/63 (3.1%) 2/63 (3.1%) 4/63 (6.2%) 9/63 (14.8%) 1/63 (1.5%) 13/63 (20.6%) 22/63 (34.9%)
Viral load < 50 copies/mL Viral load ≥ 50 copies/ml	57/63 (90.4%) 6/63 (9.6%)
CD4 > 500/μL CD4 200–500/μL CD4 < 200/μL	41/63 (66%) 15/63 (23.8%) 7/63 (10.2%)
Serological history Available anamnestic data Unavailable anamnestic data	54/63 (85.7%) 9/63 (14.3%)
Ever-positive serology HBV HCV HHV-1 HHV-2 HHV-3 HHV-8 EBV CMV HPV-HR Toxoplasma gondii Treponema pallidum	20/54 (37%) 17/54 (31.4%) 19/54 (35.1%) 8/54 (14.8%) 7/54 (12.9%) 19/54 (35.1%) 22/54 (40.7%) 26/54 (48.1%) 1/54 (1.8%) 12/54 (22.2%) 17/54 (31.4%)

**Table A3:** Distribution of AIDS-defining (ADC) and non-AIDS-defining (NADC) oncological disease types in the study sample.

**Criteria**	**No. Cases/Total (%)**
Total cancer cases	70/534 (13.1%)
ADCs NADCs	26/70 (37.1%) 44/70 (62.9%)
ADC prevalence Kaposi’s sarcomas Non-Hodgkin Lymphomas Cervical cancers	19/70 (27.1%) 6/70 (8.5%) 1/70 (1.4%)
NADC prevalence Skin cancers Head and neck cancers Thoracic cancers Breast cancers Gastrointestinal cancers Hematolymphoid system cancers Urogenital cancers	14/70 (20%) 9/70 (12.8%) 2/70 (2.8%) 7/70 (10%) 4/70 (5.7%) 2/70 (2.8%) 6/70 (8.5%)

**Table A4:** Oncologic pathology classified by ICD-10 codes, categorized by ADC and non-ADC.

AIDS Defining Cancers	ICD-10
Kaposi’s sarcoma	C46.9
Non-Hodgkin lymphoma • B-cell lymphoma • Burkitt lymphoma	• C85.10 • C83.70
Cervical cancer	C53.9
**Non-AIDS Defining Cancers**	**ICD-10**
Malignant neoplasms of lip, oral cavity and pharynx • Malignant neoplasm of oropharynx • Malignant neoplasm of nasopharynx	• C10.9 • C11.9
Malignant neoplasms of thyroid and other endocrine glands • Malignant neoplasm of thyroid gland	• C73
Malignant neoplasms of respiratory and intrathoracic organs • Malignant neoplasm of larynx • Malignant neoplasm of lung	• C32.9 • C34.90
Malignant neoplasms of breast	C50.919
Malignant neoplasms of digestive organs • Malignant neoplasm of liver • Malignant neoplasm of pancreas	• C22.9 • C25.9
Malignant neoplasms of urinary tract • Malignant neoplasm of kidney • Malignant neoplasm of bladder	• C64.9 • C67.9
Malignant neoplasms of male genital organs • Malignant neoplasm of prostate • Malignant neoplasm of testis	• C61 • C62.90
Malignant neoplasms of lymphoid, hematopoietic and related tissue • Hodgkin lymphoma	• C81.90
Melanoma and other malignant neoplasms of skin • Malignant melanoma of skin • Basal cell carcinoma of skin • Squamous cell carcinoma of skin	• C43.9 • C44.91 • C44.92
Malignant neuroendocrine tumor	C7A

**Table A5:** Statistical analysis of clinical and virological characteristics in patients with AIDS-defining (ADC) and non-AIDS-defining (NADC) neoplasms. ° Fisher’s exact test was used for 2 × 2 contingency tables, while the chi-square test was applied to all other categorical comparisons. * *p*-value < 0.

Criteria	ADC+NADC	ADC	NADC	*p*-Value ° ADC vs. NADC
Total Males Females	70 (100%) 55/70 (78.5%) 15/70 (21.5%)	26/70 (37.2%) 21/55 (38.1%) 5/15 (33.4%)	44/70 (62.8%) 34/55 (61.9%) 10/15 (66.6%)	>0.99
Italians Foreign nationals	61/70 (87.1%) 9/70 (12.8%)	22/61 (36%) 4/9 (44.4%)	39/61 (64%) 5/9 (55.6%)	0.7178
Age < 50 years Age ≥ 50 years	9/70 (12.8%) 61/70 (87.2%)	6/9 (66.6%) 20/61 (32.8%)	3/9 (33.4%) 41/61 (67.2%)	0.06846
Duration of HIV infection ≥ 20 years Duration of HIV infection < 20 years	31/70 (44.2%) 39/70 (55.8%)	8/31 (25.8%) 18/39 (46.1%)	23/31 (74.2%) 21/39 (53.9%)	0.08938
CDC A1 CDC A2 CDC A3 CDC B1 CDC B2 CDC B3 CDC C1 CDC C2 CDC C3	3/70 (4.2%) 8/70 (11.4%) 2/70 (2.8%) 2/70 (2.8%) 4/70 (5.7%) 10/70 (14.3%) 1/70 (1.4%) 16/70 (22.8%) 24/70 (34.2%)	0/3 (0%) 0/8 (0%) 1/2 (50%) 0/2 (0%) 0/4 (0%) 2/10 (20%) 0/1 (0%) 9/16 (56.2%) 14/24 (58.4%)	3/3 (100%) 8/8 (100%) 1/2 (50%) 2/2 (100%) 4/4 (100%) 8/10 (80%) 1/1 (100%) 7/16 (43.8%) 10/24 (41.6%)	0.06015
Viral load < 50 copies/mL Viral load ≥ 50 copies/mL	64/70 (91.4%) 6/70 (8.6%)	21/64 (32.8%) 5/6 (83.3%)	43/64 (67.2%) 1/6 (16.7%)	0.02383 *
CD4 > 500/μL CD4 200–500/μL CD4 < 200/μL	46/70 (65.7%) 17/70 (24.3%) 7/70 (10%)	17/46 (36.9%) 4/17 (23.5%) 5/7 (71.4%)	29/46 (63.1%) 13/17 (76.5%) 2/7 (28.6%)	0.0874
Serological history Available anamnestic data Unavailable anamnestic data	55/70 (78.5%) 15/70 (21.5%)	23/55 (41.8%) 3/15 (20%)	32/55 (58.2%) 12/15 (80%)	0.1441
Ever-positive serology HBV HCV HHV-1 HHV-2 HHV-3 HHV-8 EBV CMV HPV-HR Toxoplasma gondii Treponema pallidum	20/55 (36.3%) 17/55 (30.9%) 18/55 (32.7%) 8/55 (14.5%) 7/55 (12.7%) 19/55 (34.5%) 22/55 (40%) 26/55 (47.2%) 1/55 (1.8%) 12/55 (21.8%) 17/55 (30.9%)	5/20 (25%) 5/17 (29.4%) 6/18 (33.3%) 5/8 (62.5%) 5/7 (71.4%) 19/19 (100%) 10/22 (45.4%) 10/26 (38.4%) 0/1 (0%) 6/12 (50%) 11/17 (64.7%)	15/20 (75%) 12/17 (70.6%) 12/18 (66.7%) 3/8 (37.5%) 2/7 (28.6%) 0/19 (0%) 12/22 (54.6%) 16/26 (61.6%) 1/1 (100%) 6/12 (50%) 6/17 (35.3%)	0.0001426 *
